# Serum LEAP-2 as a Potential Biomarker for Hepatic Steatosis in Adolescents with Obesity and MASLD: A Cross-Sectional Study

**DOI:** 10.3390/diagnostics15212816

**Published:** 2025-11-06

**Authors:** Sevim Çakar, Nur Arslan, Mehmet Ateş, Oya Sayın, Oğuzhan Akyaz, Tuğçe Tatar Arık, Rabia Ilgın, Nilay Danış

**Affiliations:** 1 Division of Pediatric Gastroenterology, Department of Pediatrics, Faculty of Medicine, Dokuz Eylül University, Izmir 35340, Turkey; 2 Division of Pediatric Metabolism and Nutrition, Department of Pediatrics, Dokuz Eylül University, Izmir 35340, Turkey; 3 Vocational School of Health Services, Dokuz Eylül University, Izmir 35340, Turkey; 4 Department of Physiology, School of Medicine, Dokuz Eylül University, Izmir 35340, Turkey; 5 Department of Gastroenterology, Faculty of Medicine, Dokuz Eylül University, Izmir 35340, Turkey

**Keywords:** MASLD, hepatosteatosis, obesity, pediatrics, LEAP-2, FibroScan^®^, biomarker

## Abstract

**Background/Objectives:** Metabolic dysfunction-associated steatotic liver disease (MASLD) is becoming more common among adolescents, but non-invasive biomarkers for early detection are still limited. Liver-expressed antimicrobial peptide-2 (LEAP-2), a ghrelin receptor antagonist, has been connected to obesity and liver fat buildup in adults, but pediatric data are limited. This study investigates the hypothesis that higher levels of LEAP-2 are associated with hepatic steatosis and the role of LEAP-2 serum levels in the earlier and easier diagnosis of MASLD in children. **Methods:** In this cross-sectional study, 51 adolescents aged 12–18 were divided into three groups: one with MASLD and obesity (MASLD-Ob) (confirmed hepatosteatosis by imaging studies such as magnetic resonance or ultrasound, along with at least one cardiometabolic criterion and a body mass index (BMI) > 2 SD) (*n* = 19), another with obesity without any liver pathology or MASLD (BMI > 2 SD) (*n* = 14), and healthy controls (*n* = 18). The controlled attenuation parameter (CAP) was measured using FibroScan^®^ Mini + 430 (Echosens SA, Créteil, France), and serum ghrelin and LEAP-2 levels were determined via ELISA. Correlations between LEAP-2, ghrelin, CAP, BMI z-score, and metabolic parameters were analyzed. **Results:** LEAP-2 and ghrelin levels among the three groups were similar (*p* = 0.148, *p* = 0.515). A positive correlation was observed between LEAP-2 levels and CAP values in the obese group (both the MASLD-Ob and obesity groups) (r = 0.379, *p* = 0.030). When a cutoff of 240 dB/m was used, the median LEAP-2 level in cases above this value was 2.20 ng/mL, compared to 1.37 ng/mL in cases below it (*p* = 0.021), which was significantly different. When analyzing the obese group (both the MASLD-Ob and obese groups) a statistically significant correlation was found between serum LEAP-2 levels and CAP, AST, GGT, and total bilirubin values (r = 0.379, *p* = 0.030; r = 0.369, *p* = 0.035; r = 0.369, *p* = 0.035; r = 0.357, *p* = 0.049, respectively). **Conclusions:** Interventional imaging methods and biomarkers for diagnosing and monitoring hepatosteatosis have become well-established in the literature. However, since these tests are not available at all centers and can be costly, there is an increasing search for other easily accessible diagnostic and follow-up parameters. LEAP-2 could be a promising non-invasive biomarker for pediatric MASLD, especially when used alongside CAP measurements. The application of this biomarker in pediatric MASLD provides valuable data to help identify and monitor the condition in adolescents. We believe our study offers strong evidence to support further research and the development of drug treatments for MASLD that aim to reduce plasma LEAP-2.

## 1. Introduction

The obesity rate in children has increased to nearly 19%, while in adolescents, it is estimated to be around 20.6% [1,2]. It is important to note that obesity is a significant concern, as are its related diseases, such as metabolic dysfunction-associated steatotic liver disease (MASLD), which is also rising among children. This urgent medical issue needs attention and immediate consideration. MASLD has replaced the term known as non-alcoholic fatty liver disease (NAFLD) and is defined as the presence of hepatic steatosis (confirmed by imaging or histology) and at least one cardiometabolic risk factor, regardless of liver enzyme levels [2,3,4]. The most recent data show a prevalence of 13% for MASLD in the pediatric population and a higher prevalence of 47% among obese children, making it the leading cause of chronic liver disease in children worldwide and a major public health concern [3]. It is important to recognize that earlier-onset disease can lead to more severe illness progression [5]. If untreated, MASLD can cause severe liver damage, progress to cirrhosis, and eventually lead to hepatocellular carcinoma [4,5].

In the complex pathogenesis of hepatosteatosis, the role of the ghrelin system has attracted considerable interest recently. Hepatic fat accumulation is promoted by ghrelin through various intracellular pathways [6]. Ghrelin, an anabolic hormone, induces hepatic steatosis by increasing the activity of transcription factors and molecules involved in de novo lipogenesis and triacylglycerol synthesis, but it also acts as a hepatoprotective agent by activating mechanisms that prevent lipotoxicity [7]. In addition, studies have shown that administration of ghrelin increases body weight [8,9,10]. While many studies indicate that ghrelin promotes adiposity and NAFLD, it also seems to have a protective effect against inflammation and fibrosis [6]. Moreover, high serum ghrelin levels are associated with a lower risk of developing MASLD [6].

Liver-expressed antimicrobial peptide 2 (LEAP-2) has recently been identified as an endogenous antagonist of the growth hormone secretagogue receptor, also known as the ghrelin receptor [11,12]. LEAP-2 is associated with liver fat content, body mass index, and fasting insulin levels [13]. By inhibiting ghrelin’s action, LEAP-2 counteracts its effects on appetite regulation and hormonal secretion pathways after administration in rats and mice, significantly reducing ghrelin-induced food intake [14]. Childhood obesity is characterized by increased ghrelin activity, which results from decreased plasma levels of LEAP-2 [15]. Ghrelin reverses the activation of fibrogenesis induced by LEAP-2, and ghrelin levels are inversely related to the severity of liver fibrosis in patients with severe obesity [16]. LEAP-2 has emerged as a promising therapeutic candidate for treating obesity [14]. Additionally, in a study conducted on an animal model, LEAP-2 has shown promising results in the treatment of MASLD [13].

There are a few studies in humans and a limited number of studies in children about the role of LEAP-2 in hepatosteatosis. Clinical research is increasing to understand the role of these molecules in pathogenesis for innovations in the diagnosis and treatment of both obesity and steatosis. This study investigates the hypothesis that higher levels of LEAP-2 are associated with hepatic steatosis and the role of LEAP-2 serum levels in the earlier and easier diagnosis of MASLD in children.

## 2. Materials and Methods

### 2.1. Ethics Statement

The study received approval from the Dokuz Eylül University Non-Interventional Research Ethics Committee (approval number: 2024/26-04, date of approval: 24 July 2024). It was conducted in accordance with the principles of the Declaration of Helsinki. Written informed consent was obtained from the parents or legal guardians of all participants, and verbal assent was obtained from children when appropriate.

### 2.2. Study Design and Participants

This cross-sectional study involved 51 adolescents aged 12 to 18 years. Among them, 19 were obese (body mass index [BMI] ≥ 95th percentile for age and sex) and confirmed to have hepatosteatosis through imaging, along with at least one cardiometabolic criterion meeting the MASLD criteria, forming the “MASLD-Ob” group [3,17]. Another 14 adolescents were obese (BMI ≥ 95th percentile) but without hepatosteatosis or MASLD criteria, constituting the “obese” group. The remaining 18 participants were in the “healthy control” group, attending our outpatient clinics for routine health evaluations (e.g., complete blood count, thyroid function tests, fasting glucose, hepatitis screening), with no known liver disease.

### 2.3. FibroScan^®^ Imaging

Fatty liver and liver stiffness were assessed by a certified physician to ensure accuracy. Reliable measurements required at least 10 valid readings, with an interquartile range below 30% of the median. Hepatic fat content was measured using the controlled attenuation parameter (CAP) in decibels per meter (dB/m), while fibrosis was evaluated via liver stiffness measurement (LSM) in kilopascals (kPa) with a FibroScan^®^ device (Echosens, Paris, France) using both M and L probes, following the manufacturer’s standard protocol. Hepatosteatosis was defined as a CAP cutoff of 240 dB/m. However, it should be noted that the CAP cutoff of >240 dB/m has not been validated in children with MASLD for detecting steatosis [18].

### 2.4. Sample Collection

Venous blood samples were collected in the morning after a 12 h overnight fast. For patients, 7 mL of blood was drawn into plain tubes for routine biochemical analyses and to measure ghrelin and LEAP-2. For the control group, in addition to the blood taken for their scheduled laboratory tests, an extra 5 mL of blood was collected in a plain tube.

### 2.5. Routine Laboratory Tests

Routine laboratory evaluations included a complete blood count, fasting plasma glucose, aspartate aminotransferase (AST), alanine aminotransferase (ALT), gamma-glutamyl transferase (GGT), total cholesterol, high-density lipoprotein cholesterol (HDL-C), low-density lipoprotein cholesterol (LDL-C), and triglycerides. ALT and AST values between 5 and 45 U/L were considered normal. In cases with elevated transaminases or ultrasonographic evidence of hepatic steatosis, additional tests were performed, including total protein, albumin, total bilirubin, prothrombin time, hepatitis B and C virus serology, TORCH panel, serum copper and ceruloplasmin, serum α_1_-antitrypsin, and autoimmune antibodies (antinuclear antibody, anti–smooth muscle antibody, anti–liver-kidney microsomal type 1 antibody) to rule out infectious, metabolic, and autoimmune liver diseases.

### 2.6. Serum Processing and Storage

Blood samples were collected from all 51 adolescents in plain tubes and allowed to clot for 30 min. They were then centrifuged at 2000–3000× *g* for 15 min at +4 °C. Serum was divided into microtubes and stored at −80 °C until analysis.

### 2.7. Measurement of Serum Ghrelin and LEAP-2 Levels

Human serum ghrelin levels were measured using a commercial ELISA kit (Cat. No. ABT2121Hu; A.B.T.™, Ankara, Turkey), with a linear range of 0.16–10 ng/mL. The intra-assay and inter-assay coefficients of variation (CVs) were less than 8% and 12%, respectively. Serum LEAP-2 concentrations were determined using a commercial ELISA kit (Cat. No. E5589Hu; Bioassay Technology Laboratory, Jiaxing, China), with a linear range of 0.1–40 ng/mL. The intra-assay and inter-assay CVs were below 4.05% and 10%, respectively. Both assays were conducted following the manufacturers’ protocols, and absorbance was measured spectrophotometrically at 450 nm using a BioTek ELX800 microplate reader (BioTek Instruments, Winooski, VT, USA).

### 2.8. Statistical Analysis

All analyses were conducted using IBM SPSS Statistics for Windows, version [IBM SPSS Statistics version 27] (IBM Corp., Armonk, NY, USA). Descriptive statistics are shown as mean ± standard deviation (SD) or median (interquartile range) for continuous variables and as counts (percentages) for categorical variables. Group comparisons were performed by the Mann–Whitney U test for continuous data and the chi-square test for categorical data. Relationships between continuous variables were assessed with Spearman’s rank correlation coefficient. A *p*-value < 0.05 was considered to be statistically significant.

## 3. Results

The groups were similar in terms of sex and age distribution (Table 1). The comparison of laboratory results and FibroScan^®^ parameters among the groups is shown in Table 1 and Table 2, respectively.

At a CAP threshold of 240 dB/m (*n* = 24) and a threshold of 270 dB/m (*n* = 17), the prevalence of steatotic liver (detecting steatosis grade ≥ 1 using abdominal ultrasonography or magnetic resonance) was 70.8% and 88.2%, respectively, among overall adolescents. Two patients with CAP thresholds below 240 dB/m had steatosis in imaging, and one patient in the healthy group had a threshold above 240 dB/m. In MASLD-Ob and obese groups, participants with CAP ≥ 240 dB/m had significantly higher LEAP-2 levels (median = 2.20 ng/mL, min = 1.04-max = 5.31) than those with CAP < 240 dB/m (median = 1.37 ng/mL, min = 0.96-max = 3.11) (*p* = 0.021). In obese and MASLD-Ob groups, participants with CAP ≥ 240 dB/m had similar ghrelin levels (median = 2.12 ng/mL, min = 1.08-max = 7.69) compared to those with CAP < 240 dB/m (median = 1.81 ng/mL, min = 1.18-max = 3.90) (*p* = 0.895).

In the analysis of all patients (*n* = 51), CAP values were significantly correlated with LSM, AST, ALT, insulin, HOMA, GGT, and BMI standard deviation levels (r = 0.504, r = 0.498, r = 0.646, r = 0.586, r = 0.594, r = 0.690, and r = 0.679, all *p* < 0.001, respectively). Triglyceride and HDL-cholesterol levels also correlated with CAP levels (r = 0.594, *p* = 0.006; r = 0.301, *p* = 0.032, respectively). CAP values did not correlate with serum LEAP-2 and ghrelin concentrations (r = 0.172, *p* = 0.226 and r = −0.069, *p* = 0.630, respectively). There was no correlation between LEAP-2 or ghrelin levels and the recorded laboratory, anthropometric parameters, or FibroScan^®^ imaging records when all patients were evaluated.

In the analysis of both the MASLD-Ob and obese groups (*n* = 33), a statistically significant correlation was identified between serum LEAP-2 levels and CAP, AST, GGT, and total bilirubin values (r = 0.379, *p* = 0.030; r = 0.369, *p* = 0.035; r = 0.369, *p* = 0.035; r = 0.357, *p* = 0.049, respectively). No correlation was found between LEAP-2 levels and other laboratory and anthropometric parameters in these groups. Moreover, there was no statistically significant correlation between serum ghrelin levels and the measured laboratory, anthropometric parameters, or FibroScan^®^ Imaging records in these patients. In the analysis of both the obese and MASLD-Ob groups, CAP values were significantly correlated with the same factors of all participant groups: LSM, AST, ALT, insulin, HOMA, GGT, and BMI standard deviation levels (r = 0.440, *p* = 0.010; r = 0.662, *p* < 0.001; r = 0.765, *p* < 0.001; r = 0.448, *p* = 0.004; r = 0.497, *p* = 0.004; r = 0.778, *p* < 0.001; r = 0.440, *p* = 0.010, respectively).

## 4. Discussion

In this novel clinical study, we found that LEAP-2 (the latest key member of the ghrelin system) levels were higher in children with CAP above 240 dB/m in FibroScan^®^, which is defined as a cut-off value for hepatosteatosis [9,18]. Another noteworthy finding was that LEAP-2 levels were correlated with CAP, AST, and GGT in obese children and MASLD with obesity.

Another result that could not reach statistical significance was that low serum ghrelin levels were found in adolescents with MASLD-Ob compared to both obese and healthy peers. Similarly, decreased circulating ghrelin concentrations in relation to MASLD have been reported in recent studies [6,19,20]. On the other hand, insulin levels exhibited marked similarity between the obese and MASLD-Ob groups. Ghrelin also reflects other metabolic mechanisms that lead to lower ghrelin levels in patients with fatty liver, beyond insulin metabolism. Additionally, ghrelin influences lipid metabolism, promoting an increase in white adipose tissue mass and stimulating lipogenesis in the liver [21]. Studies on animals have demonstrated that knocking out LEAP-2 can lessen ghrelin’s effects on feeding behavior and decrease hepatocyte lipogenesis and storage. It may also worsen hepatic inflammation by blocking ghrelin’s protective effects [22]. Many studies have recognized LEAP-2 as a potential therapeutic target for ghrelin-related diseases, including obesity, cachexia, diabetes, anorexia, alcohol abuse, and Prader–Willi syndrome [8,18]. Experimental evidence indicates LEAP-2 opposes ghrelin-mediated lipogenesis and inflammation, potentially influencing disease progression [23,24].

In this cross-sectional study, we observed elevated serum LEAP-2 levels in adolescents with MASLD-Ob compared to both obese and healthy peers, although not reaching statistical significance. However, higher LEAP-2 levels were found in adolescents with CAP ≥ 240 dB/m. and LEAP-2 levels correlate with AST, GGT, and total bilirubin in the obese and MASLD-Ob groups. Interestingly, LEAP-2 shows a positive correlation with AST in our study rather than ALT, which is well-established as a biomarker for hepatocellular injury in studies [23,25,26]. Thus, aminotransferases will continue to be important in the diagnosis and research of liver injury in the future [27]. In the literature, LEAP-2 has been associated with hepatic steatosis, impacting the lipolytic/lipogenic pathway and insulin signaling in mice and humans [23]. Similarly, our study, along with those of Liu and Ma, found no correlation between triglycerides, cholesterol, and LEAP-2 [23,26].

The North American Society of Pediatric Gastroenterology, Hepatology, and Nutrition (NASPGHAN) guideline recommends considering a liver biopsy for children with an increased risk of non-alcoholic fatty liver disease or fibrosis, indicated by hepatomegaly and ALT levels exceeding 80 U/L [28]. Ma et al. suggest that a cut-off of ALT levels ≥ 80 U/L can lead to underdiagnosis of pediatric MASLD and recommend referral to hepatology at lower ALT thresholds (≥52 U/L for boys; ≥44 U/L for girls) [29]. Moreover, the most recent American Association for the Study of Liver Diseases (AASLD) practice statement, published in 2025, has stated that if persistently elevated ALT (>52 U/L for boys; >44 U/L for girls) is detected in a child, a liver biopsy should be considered to evaluate MASLD. The median ALT level of 63 (13–186) U/L in MASLD-Ob patients in our study supports this recommendation.

Liver biopsy is the current gold standard for diagnosis and prognosis, but children with severe obesity may face complications when undergoing this procedure [1,28]. The low acceptance of invasive procedures by patients has created an urgent need for reliable, accurate, and minimally invasive or non-invasive diagnostic biomarkers. Therefore, there is a requirement for non-invasive diagnostic methods that can also be used for follow-up in MASLD. The NAFLD Liver Fat Score [30], the Hepatic Steatosis Index [31], the Fatty Liver Index [32], the Visceral Adiposity Index [33], and many others are limited in their ability to detect steatosis. Levels of procollagen III can help identify patients with or without a histological diagnosis of non-alcoholic steatohepatitis (NASH), as these levels show a relatively linear relationship with the grade of NASH [34]. Alpha-2 macroglobulin, hyaluronic acid (HA), tissue inhibitor of metalloproteinase 1, and the amino-terminal peptide of procollagen III are included in a validated serum panel to distinguish mild to moderate fibrosis from advanced fibrosis in adult NAFLD patients [35]. Also, ALT and GGT are effective predictors of histological improvement in pediatric nonalcoholic steatohepatitis [25]. Several other biomarkers have been studied to distinguish NASH in children, including cathepsin D, serum cytokeratin 18, angiopoietin-2, and cytokines such as total activated plasminogen activator inhibitor 1 (PAI-1), PAI-1, IL-8, and soluble IL-2 receptor alpha (sIL2Rα) [36,37,38,39]. To date, there are no validated non-invasive serum biomarkers available to assess steatosis in children [40]. This situation also makes our clinical study on LEAP-2 in obese and healthy children, a novel biomarker, valuable.

Advanced Magnetic Resonance Imaging (MRI) and Transient Elastography (TE) are other non-invasive imaging techniques used in MASLD diagnosis [41,42]. Utilizing ultrasound as an initial imaging option in children with suspected MASLD when MRI-proton density fat fraction is unavailable remains a valid recommendation [40]. One pediatric study found that CAP had a sensitivity of 75% and a specificity of 75% at an optimal cutoff value of 277 dB/m for grade 1 steatosis in severely obese children, and CAP is not significantly more effective than ultrasound at diagnosis [43]. A recent pediatric study demonstrates much higher reliability results for TE [44,45]. In our cohort, at CAP thresholds of 240 dB and 270 dB, the prevalence of steatotic liver (detecting steatosis grade ≥ 1 via abdominal ultrasonography) was 70.8% and 88.2%, respectively. It should be noted that CAP has not been validated in pediatric populations for the level of precision required for clinical decision-making [40]. Another key finding of our study is that CAP ≥ 240 dB/m was a clear cutoff associated with higher LEAP-2 levels and correlated with LEAP-2 levels in children with CAP ≥ 240 dB/m. While CAP provides non-invasive quantification, combining it with serum LEAP-2 levels may improve early detection and monitoring strategies in MASLD. We need to emphasize that it was found that there was a significant correlation between LEAP-2 and CAP in obese adolescents, but the LEAP-2 levels between the three groups were not statistically different.

Despite a small sample size, our study’s strengths include standardized ELISA assays, blinded CAP measurements, and strict diagnostic criteria. In our study, LEAP 2 and ghrelin levels may not differ between the groups, but it would be more valuable to study their functions in vivo in tissues rather than serum levels. This is one of the most significant limitations of our study. Other limitations are its cross-sectional design and absence of histological confirmation, since liver biopsy remains the diagnostic gold standard for MASLD and liver fibrosis.

## 5. Conclusions

In this study, significant differences in LEAP-2 levels were observed in adolescents with CAP ≥ 240 dB/m. Also, serum LEAP-2 levels were higher in adolescents with MASLD and are associated with CAP, AST, GGT, and total bilirubin, which are the parameters indicating the severity of hepatic steatosis. Thus, LEAP-2 could be a promising non-invasive biomarker for pediatric MASLD, especially when used alongside CAP measurements. The application of this biomarker in pediatric MASLD provides valuable data to help identify and monitor the condition in adolescents. The small sample size causes the results to have the characteristics of preliminary study data, indicating the need for further longitudinal studies on larger cohorts with histological validation to assess LEAP-2 dynamics. We believe our study offers strong evidence to support further research and the development of drug treatments for MASLD that aim to reduce plasma LEAP-2.

## Figures and Tables

**Table 1:** Characteristics of the Demographics and Laboratory Results of the Obese MASLD (MASLD-Ob), Obese, and Healthy Controls.

	MASLD-Ob (*n* = 19)	Obese (*n* = 14)	Healthy Control (*n* = 18)	*p*
**Age year,** **median (IQR)**	14.0 (12.0, 17.0)	14.5 (12.0, 17.0)	14.5 (12.0, 17.0)	0.982
**Gender (Female),** ***n*** **(%)**	9 (47.4%)	7 (50%)	10 (55.5%)	0.898
**Weight SD, Mean ± SD**	3.23 ± 1.30	3.00 ± 0.89	−0.41 ± 1.25	<0.001
**Height SD,** **Mean ± SD**	1.08 ± 1.13	1.03 ± 0.89	−0.19 ± 1.25	0.002
**BMI SD,** **median (IQR)**	2.74 (2.01, 4.20)	2.18 (1.72, 5.10)	−0.66 (−2.10, 2.24)	<0.001
**AST IU/L,** **median (IQR)**	34.0 (16.0, 68.0)	18.0 (13.0, 61.0)	20.0 (13.0, 38.0)	0.001
**ALT IU/L,** **median (IQR)**	63.0 (13.0, 186.0)	17.0 (12.0, 51.0)	12.0 (7.0, 53.0)	<0.001
**GGT IU/L,** **median (IQR)**	32.0 (15.0, 101.5)	17.0 (13.0, 44.0)	13.0 (9.0, 24.0)	<0.001
**Glucose mg/dL, Mean ± SD**	86.3 ± 8.7	83.4 ± 7.1	83.8 ± 8	0.423
**Insulin mIU/L, median (IQR)**	27.7 (7.9, 111.8)	25.3 (5.7, 57.8)	9.6 (5.7, 29.3)	<0.001
**HOMA,** **median (IQR)**	5.8 (1.9, 28.4)	4.5 (1.2, 10.8)	2.0 (1.1, 3.7)	<0.001
**Triglyceride, mg/dL,** **median (IQR)**	121.0 (32.0, 264.0)	149.0 (74.0, 357.0)	70.5 (50.0, 143.0)	0.005
**LDL cholesterol mg/dL,** **median (IQR)**	91.5 (61.6, 617.0)	98.8 (35.6, 144.5)	83.0 (28.6, 135.3)	0.570
**HDL cholesterol mg/dL,** **median (IQR)**	38.0 (27.9, 46.3)	56.4 (34.0, 71.6)	50.8 (38.0, 68.0)	0.005
**Vitamin D mg/L, median (IQR)**	25 (9.1, 27.7)	15.3 (9.0, 28.6)	19.2 (9.5, 30.4)	0.120
**Total bilirubin, median (IQR)**	0.65 (0.43, 1.30)	0.48 (0.32, 1.71)	0.80 (0.42, 1.70)	0.194

Abbreviations: MASLD, Metabolic Dysfunction-Associated Steatotic Liver Disease; IQR, Interquartile Range; SD, Standard Deviation; BMI, Body Mass Index; ALT, alanine aminotransferase; AST, aspartate aminotransferase; GGT, gamma-glutamyl transferase; HOMA, homeostasis model assessment; LDL, low-density lipoprotein; HDL, high-density lipoprotein.

**Table 2:** FibroScan^®^ Parameters and Biomarkers in Obese, Obese MASLD (MASLD-Ob), and Healthy Controls.

	MASLD-Ob (*n* = 19)	Obese (*n* = 14)	Healthy Control (*n* = 18)	*p*
**FibroScan^®^** **CAP (dB/m),** **Mean ± SD**	286.0 ± 45.5 *	230.7 ± 41.4 *	193.6 ± 31.1 *	* <0.001
**FibroScan^®^** **LSM (kPa),** **median (IQR)**	5.8 * (4.1, 11.7)	4.4 (3.4, 6.5)	4.2 * (2.5, 6.7)	* <0.001
**LEAP-2 (ng/mL),** **median (IQR)**	2.24 (1.04, 5.31)	1.45 (0.96, 4.50)	1.77 (0.90, 4.32)	0.148
**Ghrelin (ng/mL), median (IQR)**	1.92 (1.08, 7.69)	2.43 (1.18, 3.90)	2.44 (1.08, 4.20)	0.515
**LEAP-2/Ghrelin ratio, median (IQR)**	1.2 (0.41, 2.42)	0.63 (0.34, 1.27)	0.79 (0.32, 2.68)	0.129

Abbreviations: MASLD, Metabolic Dysfunction-Associated Steatotic Liver Disease; CAP, controlled attenuation parameter; LSM, liver stiffness measurement; LEAP-2, liver-expressed antimicrobial peptide 2; * indicates groups with significant differences.

## Data Availability

The data sets supporting the conclusions of this article are not publicly accessible (for legal and ethical reasons) but can be obtained from the corresponding author upon reasonable request.

## Abbreviations

The following abbreviations are used in this manuscript:

MASLD	Metabolic dysfunction-associated steatotic liver disease
LEAP-2	Liver-expressed antimicrobial peptide-2
NAFLD	Non-alcoholic fatty liver disease
BMI	Body mass index
CAP	Controlled attenuation parameter
LSM	Liver stiffness measurement
AST	Aspartate aminotransferase
ALT	Alanine aminotransferase
GGT	Gamma-glutamyl transferase
HDL-C	High-density lipoprotein cholesterol
LDL-C	Low-density lipoprotein cholesterol
CVs	Coefficients of variation
SD	Standard deviation
HOMA	Homeostasis model assessment
NASPGHAN	The North American Society of Pediatric Gastroenterology, Hepatology, and Nutrition
AASLD	Association for the Study of Liver Diseases
NASH	Non-alcoholic steatohepatitis
PAI-1	plasminogen activator inhibitor 1
MRI	Magnetic Resonance Imaging
TE	Transient Elastography

## References

[1] Panganiban J., Kehar M., Ibrahim S.H., Hartmann P., Sood S., Hassan S., Ramirez C.M., Kohli R., Censani M., Mauney E. (2025). Metabolic dysfunction-associated steatotic liver disease (MASLD) in children with obesity: An Obesity Medicine Association (OMA) and expert joint perspective 2025. Obes. Pillars.

[2] Hampl S.E., Hassink S.G., Skinner A.C., Armstrong S.C., Barlow S.E., Bolling C.F., Avila Edwards K.C., Eneli I., Hamre R., Joseph M.M. (2023). Clinical practice guideline for the evaluation and treatment of children and adolescents with obesity. Pediatrics.

[3] Xanthakos S.A., Ibrahim S.H., Adams K., Kohli R., Sathya P., Sundaram S., Vos M.B., Dhawan A., Caprio S., Behling C.A. (2025). AASLD practice statement on the evaluation and management of metabolic dysfunction-associated steatotic liver disease in children. Hepatology.

[4] Rinella M.E., Lazarus J.V., Ratziu V., Francque S.M., Sanyal A.J., Kanwal F., Romero D., Abdelmalek M.F., Anstee Q.M., Arab J.P. (2024). A multisociety Delphi consensus statement on new fatty liver disease nomenclature. Ann. Hepatol..

[5] Ha S., Wong V.W., Zhang X., Yu J. (2024). Interplay between gut microbiome, host genetic and epigenetic modifications in MASLD and MASLD-related hepatocellular carcinoma. Gut.

[6] Quiñones M., Fernø J., Al-Massadi O. (2020). Ghrelin and liver disease. Rev. Endocr. Metab. Disord..

[7] Tuero C., Becerril S., Ezquerro S., Neira G., Frühbeck G., Rodríguez A. (2023). Molecular and cellular mechanisms underlying the hepatoprotective role of ghrelin against NAFLD progression. J. Physiol. Biochem..

[8] Müller T.D., Nogueiras R., Andermann M.L., Andrews Z.B., Anker S.D., Argente J., Batterham R.L., Benoit S.C., Bowers C.Y., Broglio F. (2015). Ghrelin. Mol. Metab..

[9] Gupta D., Ogden S.B., Shankar K., Varshney S., Zigman J.M. (2021). A LEAP2 conclusion? Targeting the ghrelin system to treat obesity and diabetes. Mol. Metab..

[10] Mani B.K., Zigman J.M. (2017). Ghrelin as a survival hormone. Trends Endocrinol. Metab..

[11] Huang X., Deng Z., Li X., Yan S., Zhong K., Yuan F., Liu L., Deng C., Liu T., Zhao R. (2025). Serum LEAP2 levels across the spectrum of metabolic dysfunction-associated fatty liver disease: A potential noninvasive biomarker for severity stratification. Diabetes Metab. Syndr. Obes..

[12] Shankar K., Metzger N.P., Lawrence C., Gupta D., Osborne-Lawrence S., Varshney S., Singh O., Richard C.P., Zaykov A.N., Rolfts R. (2024). A long-acting LEAP2 analog reduces hepatic steatosis and inflammation and causes marked weight loss in mice. Mol. Metab..

[13] Lin C.E., Chen C.Y. (2024). Impacts of central administration of the novel peptide, LEAP-2, in different food intake models in conscious rats. Nutrients.

[14] Lugilde J., Casado S., Beiroa D., Cuñarro J., Garcia-Lavandeira M., Álvarez C.V., Nogueiras R., Diéguez C., Tovar S. (2022). LEAP-2 counteracts ghrelin-induced food intake in a nutrient, growth hormone and age independent manner. Cells.

[15] Fittipaldi A.S., Hernández J., Castrogiovanni D., Lufrano D., De Francesco P.N., Garrido V., Vitaux P., Fasano M.V., Fehrentz J.-A., Fernández A. (2020). Plasma levels of ghrelin, des-acyl ghrelin and LEAP2 in children with obesity: Correlation with age and insulin resistance. Eur. J. Endocrinol..

[16] Ezquerro S., Tuero C., Becerril S., Valentí V., Moncada R., Landecho M.F., Catalán V., Gómez-Ambrosi J., Mocha F., Silva C. (2023). Antagonic effect of ghrelin and LEAP-2 on hepatic stellate cell activation and liver fibrosis in obesity-associated nonalcoholic fatty liver disease. Eur. J. Endocrinol..

[17] Rinella M.E., Neuschwander-Tetri B.A., Siddiqui M.S., Abdelmalek M.F., Caldwell S., Barb D., Kleiner D.E., Loomba R. (2023). AASLD practice guidance on the clinical assessment and management of nonalcoholic fatty liver disease. Hepatology.

[18] Ge X., Yang H., Bednarek M.A., Galon-Tilleman H., Chen P., Chen M., Lichtman J.S., Wang Y., Dalmas O., Yin Y. (2018). LEAP2 is an endogenous antagonist of the ghrelin receptor. Cell Metab..

[19] Marchesini G., Pagotto U., Bugianesi E., De Iasio R., Manini R., Vanni E., Pasquali R., Melchionda N., Rizzetto M. (2003). Low ghrelin concentrations in nonalcoholic fatty liver disease are related to insulin resistance. J. Clin. Endocrinol. Metab..

[20] Arslan N., Sayin O., Tokgoz Y. (2014). Evaluation of serum xenin and ghrelin levels and their relationship with nonalcoholic fatty liver disease and insulin resistance in obese adolescents. J. Endocrinol. Investig..

[21] Lv Y., Liang T., Wang G., Li Z. (2018). Ghrelin, a gastrointestinal hormone, regulates energy balance and lipid metabolism. Biosci. Rep..

[22] Lu X., Huang L., Huang Z., Feng D., Clark R.J., Chen C. (2021). LEAP-2: An emerging endogenous ghrelin receptor antagonist in the pathophysiology of obesity. Front. Endocrinol..

[23] Ma X., Xue X., Zhang J., Liang S., Xu C., Wang Y., Zhu J. (2020). Liver expressed antimicrobial peptide 2 is associated with steatosis in mice and humans. Exp. Clin. Endocrinol. Diabetes.

[24] Hagemann C.A., Jensen M.S., Holm S., Gasbjerg L.S., Byberg S., Skov-Jeppesen K., Hartmann B., Holst J.J., Dela F., Vilsbøll T. (2022). LEAP2 reduces postprandial glucose excursions and ad libitum food intake in healthy men. Cell Rep. Med..

[25] Newton K.P., Lavine J.E., Wilson L., Behling C., Vos M.B., Molleston J.P., Rosenthal P., Miloh T., Fishbein M.H., Jain A.K. (2021). Alanine aminotransferase and gamma-glutamyl transpeptidase predict histologic improvement in pediatric nonalcoholic steatohepatitis. Hepatology.

[26] Liu Z., Ren Q., Mu H., Zeng Y., An Z., He H. (2024). Preliminary study on the diagnostic value of LEAP-2 and CK18 in biopsy-proven MAFLD. BMC Gastroenterol..

[27] McGill M.R. (2016). The past and present of serum aminotransferases and the future of liver injury biomarkers. EXCLI J..

[28] Vos M.B., Abrams S.H., Barlow S.E., Caprio S., Daniels S.R., Kohli R., Mouzaki M., Sathya P., Schwimmer J.B., Sundaram S.S. (2017). NASPGHAN clinical practice guideline for the diagnosis and treatment of nonalcoholic fatty liver disease in children. J. Pediatr. Gastroenterol. Nutr..

[29] Ma N., Bansal M., Chu J., Branch A.D. (2024). Fibrosis and steatotic liver disease in US adolescents according to the new nomenclature. J. Pediatr. Gastroenterol. Nutr..

[30] Kotronen A., Peltonen M., Hakkarainen A., Sevastianova K., Bergholm R., Johansson L.M., Lundbom N., Rissanen A., Ridderstråle M., Groop L. (2009). Prediction of non-alcoholic fatty liver disease and liver fat using metabolic and genetic factors. Gastroenterology.

[31] Lee J.H., Kim D., Kim H.J., Lee C.H., Yang J.I., Kim W., Kim Y.J., Yoon J.-H., Cho S.-H., Sung M.-W. (2010). Hepatic steatosis index: A simple screening tool reflecting nonalcoholic fatty liver disease. Dig. Liver Dis..

[32] Bedogni G., Bellentani S., Miglioli L., Masutti F., Passalacqua M., Castiglione A., Tiribelli C. (2006). The fatty liver index: A simple and accurate predictor of hepatic steatosis in the general population. BMC Gastroenterol..

[33] Pascot A., Lemieux S., Lemieux I., Prud’Homme D., Tremblay A., Bouchard C., Nadeau A., Couillard C., Tchernof A., Bergeron J. (1999). Age-related increase in visceral adipose tissue and body fat and the metabolic risk profile of premenopausal women. Diabetes Care.

[34] Nielsen M.J., Nedergaard A.F., Sun S., Veidal S.S., Larsen L., Zheng Q., Suetta C., Henriksen K., Christiansen C., Karsdal M.A. (2013). The neo-epitope specific PRO-C3 ELISA measures true formation of type III collagen associated with liver and muscle parameters. Am. J. Transl. Res..

[35] Loomba R., Jain A., Diehl A.M., Guy C.D., Portenier D., Sudan R., Singh S., Faulkner C., Richards L., Hester K.D. (2019). Validation of serum test for advanced liver fibrosis in patients with nonalcoholic steatohepatitis. Clin. Gastroenterol. Hepatol..

[36] Fitzpatrick E., Mitry R.R., Quaglia A., Hussain M.J., de Bruyne R., Dhawan A. (2010). Serum levels of CK18 M30 and leptin are useful predictors of steatohepatitis and fibrosis in paediatric NAFLD. J. Pediatr. Gastroenterol. Nutr..

[37] Walenbergh S.M., Houben T., Hendrikx T., Jeurissen M.L.J., van Gorp P.J., E Vreugdenhil A.C., Adriaanse M.P., A Buurman W., Hofker M.H., Mosca A. (2015). Plasma cathepsin D levels: A novel tool to predict pediatric hepatic inflammation. Am. J. Gastroenterol..

[38] Manco M., Panera N., Crudele A., Braghini M.R., Bianchi M., Comparcola D., De Vito R., Maggiore G., Alisi A. (2022). Angiopoietin-2 levels correlate with disease activity in children with nonalcoholic fatty liver disease. Pediatr. Res..

[39] Perito E.R., Ajmera V., Bass N.M., Rosenthal P., Lavine J.E., Schwimmer J.B., Yates K.P., Diehl A.M., Molleston J.P., Murray K.F. (2017). Association between cytokines and liver histology in children with nonalcoholic fatty liver disease. Hepatol. Commun..

[40] Abdelhameed F., Kite C., Lagojda L., Dallaway A., Chatha K.K., Chaggar S.S., Dalamaga M., Kassi E., Kyrou I., Randeva H.S. (2024). Non-invasive scores and serum biomarkers for fatty liver in the era of metabolic dysfunction-associated steatotic liver disease (MASLD): A comprehensive review from NAFLD to MAFLD and MASLD. Curr. Obes. Rep..

[41] Piazzolla V.A., Mangia A. (2020). Noninvasive diagnosis of NAFLD and NASH. Cells.

[42] Desai N.K., Harney S., Raza R., Al-Ibraheemi A., Shillingford N., Mitchell P.D., Jonas M.M. (2016). Comparison of controlled attenuation parameter and liver biopsy to assess hepatic steatosis in pediatric patients. J. Pediatr..

[43] Runge J.H., Van Giessen J., Draijer L.G., Deurloo E.E., Smets A.M.J.B., Benninga M.A., Koot B.G.P., Stoker J. (2021). Accuracy of controlled attenuation parameter compared with ultrasound for detecting hepatic steatosis in children with severe obesity. Eur. Radiol..

[44] Dai Kwon Y., Ko K.O., Lim J.W., Cheon E.J., Song Y.H., Yoon J.M. (2019). Usefulness of transient elastography for non-invasive diagnosis of liver fibrosis in pediatric non-alcoholic steatohepatitis. J. Korean Med. Sci..

[45] Chen B.R., Pan C.Q. (2022). Non-invasive assessment of fibrosis and steatosis in pediatric non-alcoholic fatty liver disease. Clin. Res. Hepatol. Gastroenterol..

[46] (2025). ESPGHAN 57th Annual Meeting Abstracts. JPGN Rep..

